# Evaluation of Ulnar Setting of Volar Locking Plates for Distal Radius Fractures Using Modified Skyline View

**DOI:** 10.1016/j.jhsg.2022.09.002

**Published:** 2022-10-08

**Authors:** Akane Ariga, Hidetsugu Suzuki, Kazuyuki Fukushima, Koji Fujita

**Affiliations:** ∗ Department of Orthopedic Surgery, Saku Central Hospital Advanced Care Center, Saku-City, Nagano, Japan; † Department of Orthopaedic Surgery, Dokkyo Medical University Saitama Medical Center, Saitama, Japan; ‡ Department of Functional Joint Anatomy, Graduate School of Medical and Dental Sciences, Tokyo Medical and Dental University, Tokyo, Japan

**Keywords:** Bone plate, Radiography, Radius fractures, Wrist joint

## Abstract

**Purpose:**

Volar lunate facet fragments in distal radius fractures are located at the center of the load in the wrist joint and, therefore, should be properly supported by the distal ulnar setting of volar locking plates to obtain better postoperative outcomes. This study evaluated the usefulness of the modified skyline view (MSV) in intraoperative fluoroscopy for the ulnar setting of volar locking plates by comparing it with that of the anteroposterior view (APV).

**Methods:**

Sixty-five patients with distal radius fractures who underwent open reduction and plate fixation as well as follow-up intraoperative fluoroscopy and postoperative computed tomography (CT) at our institution between April 2019 and March 2022 were included. The distance between the ulnar edge of the plate and the distal radius (d-value) was measured retrospectively using intraoperative fluoroscopy (distance measured from MSV and distance measured from APV) or postoperative CT (distance measured using postoperative CT). The distance measured from MSV and that measured from APV were compared with those measured using CT as the true values. Each measurement was performed twice by 2 examiners at an interval of 1 month. The comparison scores were evaluated using the intraclass correlation coefficient.

**Results:**

The distance measured from MSV showed a difference of 0.6 ± 0.5 mm from that measured using CT, which was significantly smaller than that measured from APV (1.2 ± 0.9 mm; *P <* .001). Neither postoperative volar subluxation nor dislocation of bone fragments was found during the study period. Both intraclass correlation coefficient (1,1) and intraclass correlation coefficient (2,1) reliabilities were substantial to almost perfect.

**Conclusions:**

Modified skyline view is an effective and versatile imaging method for estimating the ulnar setting of volar locking plates; it provides measurements more similar to those provided by postoperative CT compared with APV. The use of MSV may reduce postoperative complications, such as volar subluxation and dislocation of bone fragments, especially in cases with volar lunate facet fragments in which the ulnar setting of the plate is significant.

**Type of study/level of evidence:**

Diagnostic III.

In recent years, the importance of reduction and fixation in marginal fractures of the distal radius has been discussed. In particular, Harness et al^1^ reported that it was important to support volar lunate facet (VLF) fragments located at the ulnar edge of the distal radius and that VLF fragments were located at the center of the stability of the radiocarpal joint and distal radioulnar joint (DRUJ). The displacement of VLF fragments may lead to postoperative volar subluxation of the carpal bones, which may require secondary surgery.^1^, ^2^, ^3^, ^4^ Rikli et al^5^ demonstrated in a biomechanical study that VLF fragments are also located at the center of the load in the wrist joint.

Increasing the plate coverage of these fragments has been reported to be important for preventing the loss of reduction after operative fixation of the VLF fragments.^6^ Because VLF fragments are located at the ulnar edge of the distal radius, the plate should be placed as distally and close to the ulnar as possible. This concept was supported by an anatomic study that demonstrated that VLF fragments have an avulsion force in the radioulnar direction via the triangular fibrocartilage complex.^7^ In addition, there are several case reports in which VLF fragments were dislocated before surgery to the ulnar side rather than to the volar side.^3^^,^^8^ Therefore, in line with this concept, at our institution, we set volar locking plates as distally and close to the ulnar as possible for distal radial fractures with VLF fragments. Intraoperative imaging evaluation was, thus, suggested to be essential because the precise position of reduction directly affects postoperative outcomes.

The skyline view is an imaging method proposed by Jacob and Clay^9^ in which images are taken in the axial direction of the elbow in 75°–80° flexion and the wrist in maximum volar flexion. This view has been reported to prevent screw penetration into the dorsal cortex or DRUJ because of its clear axial view.^10^ Subsequently, several variants of the “modified” skyline view (MSV) have been reported. Marsland et al^11^ reported the “carpal shoot through view,” in which the patient’s forearm is supinated, the elbow flexed to approximately 70°, and the wrist dorsiflexed. They concluded that the carpal shoot through view could reduce the risk of postoperative pain and extensor tendon injury and help visualize the DRUJ clearly. Lee et al^12^ introduced the “radial groove view,” in which the patient’s shoulder is abducted to 90°, the elbow flexed with the forearm in neutral rotation, and the wrist flexed almost fully. They demonstrated that this radiologic method was effective for assessing the possible protrusion of screws into the groove of the extensor pollicis longus. Ozer and Toker^13^ demonstrated the “dorsal tangential view,” in which the patient’s wrist is flexed to 75° with the forearm placed between the 2 ends of the mini C-arms in the transverse plane. Furthermore, Joseph and Harvey^14^ demonstrated the “dorsal horizontal view,” in which the wrist of the patient is hyperflexed and the beam of the fluoroscopy unit aimed along the longitudinal axis of the radius. Therefore, the majority of previous reports mainly discussed the utility of MSV for confirming screw penetration into the dorsal cortex or DRUJ.

The anteroposterior view (APV) is commonly used for intraoperative fluoroscopy of volar locking plates. At our institution, the carpal shoot through view, one of the MSVs, is additionally used (Fig. 1).^11^ We found that the use of MSV in addition to APV makes the ulnar setting of volar locking plates more obvious. Therefore, we hypothesized that MSV outperformed APV in evaluating the ulnar setting of volar locking plates during intraoperative fluoroscopy. The purpose of this study was to investigate the usefulness of MSV in evaluating the ulnar setting of volar locking plates for distal radius fractures in comparison with that of APV.

**Figure 1:**
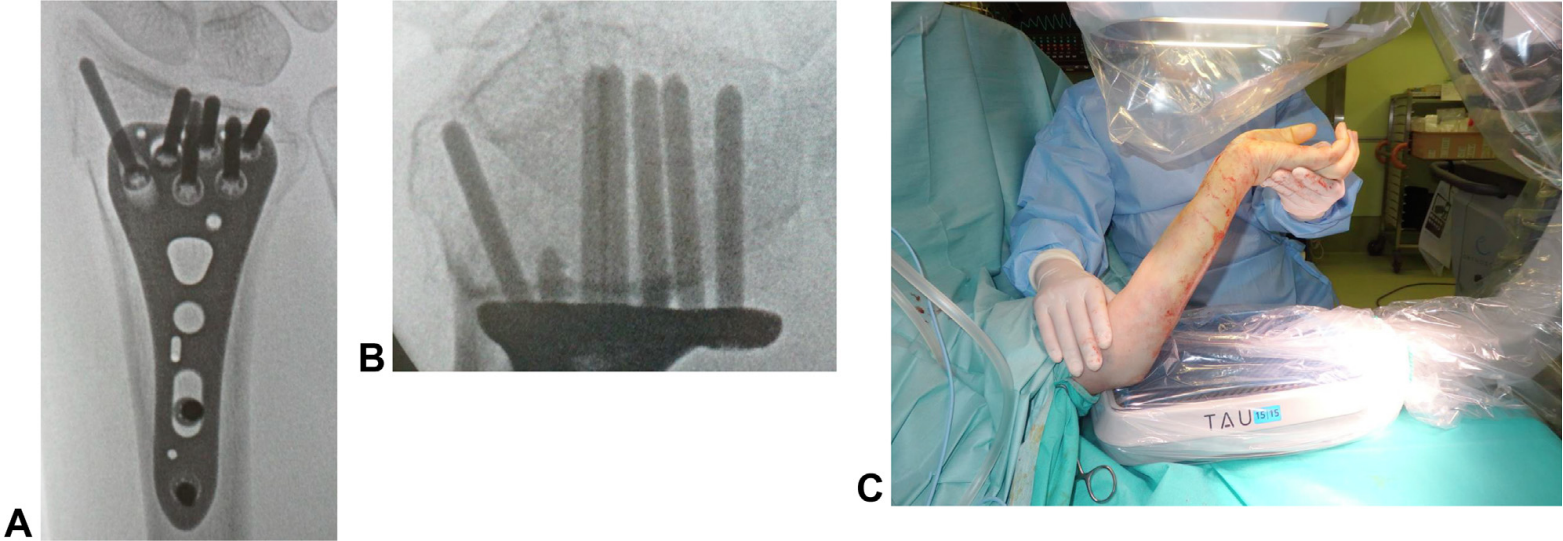
Intraoperative fluoroscopy. **A** APV after performing plate fixation with screws. **B** MSV after performing plate fixation with screws. **C** Position for obtaining MSV.

## Materials and Methods

This was a retrospective study that adhered to the tenets of the Declaration of Helsinki and was approved by the institutional review board (research protocol identification number: R202202-12). Written informed consent was obtained from all patients who participated in the study.

### Patients

Patients who had been diagnosed with a distal radial fracture, underwent open reduction and plate fixation at our institution between April 2019 and March 2022, and underwent follow-up intraoperative fluoroscopy and postoperative computed tomography (CT) were included in this study.

### Primary outcome

**Figure 2:**
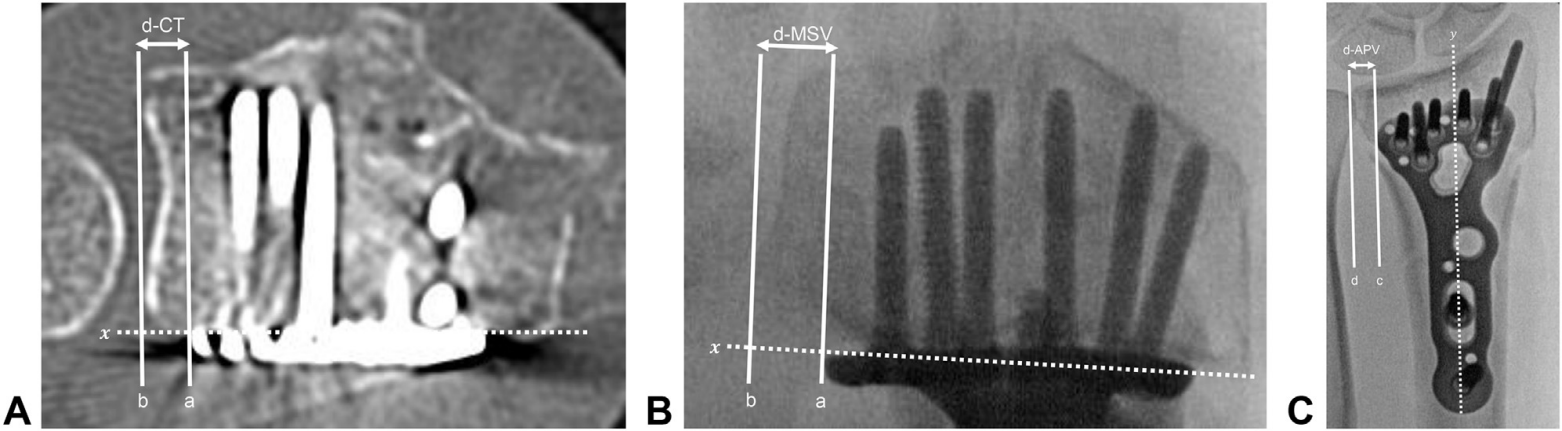
Measurement of d-values. **A** and **B** The white dotted line (x) in each image shows the line parallel to the volar edge of the distal radius. The distances between the 2 parallel lines are perpendicular to x, one of which is passing through the most ulnar edge of the volar locking plate (line “a”) and the other through the volar edge of the DRUJ (line “b”), were defined as d-CT and d-MSV, respectively. **C** The white dotted line (y) corresponds to the longitudinal axis of the radius. The distances between the 2 parallel lines perpendicular to y, one of which is passing through the most ulnar edge of the volar locking plate (line c) and the other through the volar edge of the DRUJ (line d), were defined as d-APV.

The distance between the ulnar edge of the plate and the distal radius (d-value) was measured using intraoperative fluoroscopy (distance measured from MSV [d-MSV] and distance measured from APV [d-APV]) or postoperative CT (distance measured using CT [d-CT]). The d-MSV and d-APV values were compared with the d-CT values as the true values. The difference among d-MSV, d-APV, and d-CT was set as the primary outcome. The measurements of the d-values were performed before surgery based on the intraoperative fluoroscopy (Fig. 1A, B) and postoperative CT images (Fig. 2A) by 2 hand surgeons (A.A. and H.S.) who were blinded to the radiographs and clinical data. Both the examiners received the same instructions regarding the method of measurement of the d-values to acquire data in a standard manner. The Synapse picture archiving and communication system (Fujifilm, Tokyo, Japan) was used to measure all the d-values for intraoperative fluoroscopy and postoperative CT. Both the examiners performed the measurements twice at an interval of 1 month. The reliability of the d-values was evaluated using the intraclass correlation coefficient (ICC).

### Surgical procedure

All operations were performed using the transflexor carpi radialis approach. Furthermore, MSV and APV were initiated by temporary fixation of volar locking plates.^11^ We then used forceps to crimp the plate and distal radius and inserted a cortical screw into a proximal hole. Before inserting locking screws into distal holes, a K-wire was inserted into the distal ulnar hole to check the position of the screw. In this step, MSV was frequently used. After checking if the position of the K-wire was acceptable, we moved to distal screw insertion. After the plate and screws were set in place, APV and MSV were finally checked using intraoperative fluoroscopy (Fig. 1).

### Measurement methods

#### d-CT

A straight line (x) was drawn parallel to the volar edge of the distal radius on an axial image, with the most distal end of the volar locking plate without the lunate fossa. The distance between the 2 parallel lines perpendicular to x, one of which was passing through the most ulnar edge of the volar locking plate (line “a”) and the other through the volar edge of the DRUJ (line “b”), was defined as d-CT (Fig. 2A).

#### d-MSV

A straight line (x) was drawn parallel to the volar edge of the distal radius on an axial image. The distance between the 2 lines perpendicular to x, one of which was passing through the most ulnar edge of the volar locking plate (line “a”) and the other through the volar edge of the DRUJ (line “b”), was defined as d-MSV (Fig. 2B). Because a digital scale was not available for intraoperative imaging, the screw diameter was used as a standard measurement for calibration.

#### d-APV

The long axis of the radial shaft (y) was drawn. The distance between the 2 lines parallel to y, one of which was passing through the most ulnar edge of the volar locking plate and the other through the distal ulnar edge of the volar radius, was defined as d-APV (Fig. 2C). Because a digital scale was not available for intraoperative imaging, the screw diameter was used as a standard measurement for calibration.

### Statistical analysis

The Wilcoxon matched-pairs signed-rank test was used to compare the d-values. The intrarater reliability values (2 examiners, interrater reliability values [ICC (1,1)], and interrater reliability values [ICC (2,1)]) for ICC were evaluated. JMP, version 14 (SAS Institute), was used for the statistical analysis, and *P* values <.05 were considered statistically significant. All data are presented as mean ± SD.

## Results

### Patient characteristics

There were 131 patients who were diagnosed with a distal radius fracture and underwent open reduction and plate fixation at our institution between April 2019 and March 2022. Patients who underwent follow-up intraoperative fluoroscopy and postoperative CT were included in this study. We excluded patients whose intraoperative fluoroscopy or postoperative CT images were lost. Finally, a total of 65 patients, including 52 women and 13 men with an average age of 65.8 ± 15.8 years, were identified.

### d-values

The mean d-CT was 3.3 ± 1.8 mm, the mean d-MSV was 3.3 ± 1.9 mm, and the mean d-APV was 2.7 ± 2.0 mm. The difference between d-MSV and d-CT was 0.6 ± 0.5 mm, which was significantly smaller than the difference between d-APV and d-CT (1.2 ± 0.9 mm; *P <* .001) (Table 1).

**Table 1:** Comparison of d-CT and d-MSV or d-CT and d-APV∗

	Total (n = 65)	*P* Value
d-CT and d-MSV	0.6 ± 0.5	<.001
d-CT and d-APV	1.2 ± 0.9	

∗ Data are presented as mean ± SD.

The results of ICC (1,1) and ICC (2,1) indicated that all the d-values had the reliability of substantial to almost perfect (Table 2).^15^

**Table 2:** ICCs for d-CT, d-MSV, and d-APV

	ICC (1,1)		ICC (2,1)
	Examiner 1	Examiner 2	
d-CT	0.991 (0.984–0.995)	0.961 (0.926–0.980)	0.941 (0.890–0.969)
d-MSV	0.931 (0.878–0.962)	0.945 (0.898–0.971)	0.901 (0.820–0.947)
d-APV	0.770 (0.617–0.867)	0.897 (0.812–0.945))	0.713 (0.515–0.839)

## Discussion

In the current study, we defined d-value as the distance between the ulnar edge of volar locking plates and the ulnar edge of the distal radius, which we used as a parameter for the evaluation of the distal ulnar setting of volar locking plates. The difference between d-MSV and d-CT was significantly smaller than that between d-APV and d-CT, which suggests that intraoperative MSV was better than intraoperative APV for approximating d-values measured using postoperative CT. The distal ulnar setting of volar locking plates increases plate coverage and has been reported to be important, especially for distal radius fractures with VLF fragments.^6^ Therefore, our study implied that the combined use of MSV and APV will contribute to a decrease in postoperative risks such as volar subluxation of the carpal bones induced by the loss of reduction of VLF fragments. In fact, no postoperative complications, such as volar or ulnar dislocation of bone fragments, were observed within the study period.

Because Jacob and Clay^9^ first reported the skyline view, several variants of MSV have been reported.^13^^,^^16^^,^^17^ However, as described in the introduction, most of the previous reports of MSV mainly focused on checking screw penetration into the DRUJ or dorsal cortex. We demonstrated that MSV could also be applied to the evaluation of the distal ulnar setting of volar locking plates.

The mean d-value was 3.3 ± 1.8 mm using CT, 3.3 ± 1.9 mm when measured from MSV, and 2.7 ± 2.0 mm when measured from APV; the values were smaller for APV than for MSV or CT, which implies that the use of APV may underestimate the actual d-values. Because the ulnar edge of the distal radius has various variations and the volar ulnar corner angles vary with DRUJ type, this cannot be assessed accurately using APV alone.^7^^,^^18^^,^^19^ Combining MSV with APV would allow us to set plates more precisely on the distal side of the ulnar.

It should be noted that the difference between d-MSV and d-APV was as small as approximately 0.6 mm. Patients who experienced a loss of reduction of >2 mm in ulnar variance from immediately after surgery to final follow-up were complicated by VLF fragments with a mean transverse length of 9.0 ± 3.6 mm.^20^ Obata et al^3^ also reported a case of a postoperative ulnar dislocation due to a VLF fragment with a transverse diameter of 8 mm. Moreover, Izawa et al^6^ concluded that the plate coverage against the VLF should be >65% to prevent failure. Considering these reports, the smaller the VLF bone fragments, the more precise plate placement is required. Even a difference of only 0.6 mm in the d-values can increase plate coverage by 10% for VLF fragments if the transverse length is approximately 6 mm, which poses the risk of loss of reduction after fixation. Therefore, the significant difference between d-MSV and d-APV may be clinically important, even though the difference is small.

This study has several limitations. First, we did not evaluate clinical outcomes. Second, this was a retrospective study conducted with a relatively small number of patients from a single center. In order to draw safer conclusions, more patients should be examined. Finally, we evaluated the still images using intraoperative fluoroscopy after surgery. Continuous intraoperative fluoroscopy may allow a more detailed evaluation of APV.

We hypothesized that MSV could be applied to the evaluation of the distal ulnar setting of volar locking plates. In conclusion, MSV was an effective imaging method for estimating the ulnar setting of volar locking plates; the measurement of d-MSV was significantly closer to that of d-CT than to that of d-APV. The combined use of MSV and APV during surgery may reduce postoperative complications such as volar subluxation and dislocation of bone fragments, especially in cases with VLF fragments in which the ulnar setting of the plate is significant. Further studies with a larger number of cases focusing on the relationship between MSV and postoperative clinical outcomes are expected.

## Supplementary Data

STROBE Statement
